# Diet and exercise in uterine cancer survivors (DEUS pilot) - piloting a healthy eating and physical activity program: study protocol for a randomized controlled trial

**DOI:** 10.1186/s13063-016-1260-1

**Published:** 2016-03-10

**Authors:** Dimitrios A. Koutoukidis, Rebecca J. Beeken, Ranjit Manchanda, Matthew Burnell, M. Tish Knobf, Anne Lanceley

**Affiliations:** Department of Women’s Cancer, EGA Institute for Women’s Health, University College London, London, UK; Health Behaviour Research Centre, Department of Epidemiology & Public Health, University College London, London, UK; Department of Gynaecological Oncology, Barts Health NHS Trust, Royal London Hospital, London, UK; Barts Cancer Institute, Queen Mary University of London, London, UK; Acute Care/Health Systems Division, Yale University School of Nursing, New Haven, CT USA

**Keywords:** Endometrial cancer, survivorship, behaviour change, healthy eating, physical activity

## Abstract

**Background:**

Endometrial cancer survivors comprise a high-risk group for obesity-related comorbidities. Healthy eating and physical activity can lead to better health and well-being, but this population may experience difficulties adopting healthy lifestyle practices. Personalised behaviour change programmes that are feasible, acceptable and cost-effective are needed. The aim of this trial is to pilot a manualised programme about healthy eating and physical activity.

**Methods/design:**

This is a phase II, individually randomized, parallel, controlled, two-site, pilot clinical trial. Adult endometrial cancer survivors (n = 64) who have been diagnosed with endometrial cancer within the previous 3 years and are not on active treatment will be invited to participate. Participants will be assigned in a 1:1 ratio through minimisation to either an 8-week, group-based, behaviour-change programme with weekly 90-min sessions about healthy eating and physical activity or usual care. The intervention will focus on self-monitoring, goal setting and self-rewards. Follow-up assessments will be conducted at 8 and 24 weeks from the baseline assessment. Primary feasibility outcomes will include rates of recruitment, adherence, and retention.

**Discussion:**

The study results will inform the development of a definitive randomised controlled trial to test if the programme can improve the health and quality of life of this population. It will also provide guidance on costing the intervention and the health care resource use in this population.

**Trial Registration:**

ClinicalTrials.gov identifier: NCT02433080, 20 April 2015.

**Electronic supplementary material:**

The online version of this article (doi:10.1186/s13063-016-1260-1) contains supplementary material, which is available to authorized users.

## Background

Endometrial cancer is the most common gynaecological cancer in developed countries, with more than 75 % of the patients surviving for at least 5 years [1]. Low physical activity, poor diet and obesity are risk factors for the development of endometrial cancer [2]. These factors may also be linked with quality of life after cancer treatment [3]. While the evidence on the impact of post-diagnosis health behaviours on endometrial cancer survival is still unclear [4], evidence from other cancer sites suggests that similar factors that affect cancer development may also influence survival [5–8]. However, adherence to lifestyle recommendations [9] is limited [10], putting survivors at high risk for other chronic diseases.

Cancer survivors often report making health behaviour changes [11]. As survivors might be motivated to practice health-protecting behaviours, a cancer diagnosis has been posited to be a ‘teachable moment’. However, survivors rarely initiate lifestyle behavioural changes [12–15]. Behaviour change interventions capitalising on the ‘teachable moment’ might therefore be more effective than those targeting the general population [16, 17]. While the optimal timing of the teachable moment has not been defined, the post-treatment period seems optimal for the provision of healthy eating interventions [18]. Promotion of physical activity might be independent of timing [18], but 10 physical activity trials have accrued survivors within a median of 3.1 years after their diagnosis [19]. However, willingness to participate in health behaviour trials reduces with time since diagnosis in long-term cancer survivors (>5 years since diagnosis) [20]. Qualitative data in endometrial cancer survivors also support the post-treatment period as the most appropriate time to intervene [Koutoukidis et al: Attitudes, challenges, and needs about diet and physical activity in endometrial cancer survivors: a qualitative study. Submitted]. While in a vital position to do so, health professionals do not tend to provide lifestyle advice [21], particularly given time constraints. Therefore, feasible and effective interventions are needed to promote implementation of lifestyle recommendations.

### Rationale

Theory-based behaviour change interventions suggest that improving diet and physical activity is safe, acceptable and feasible with promising effects on both psychological and physiological outcomes, including quality of life [22–25]. In the United States, the two interventions in endometrial cancer survivors have also shown sustained changes in behavioural outcomes and indicated potential changes in quality of life domains [26—27]. Quality of life and its components are valuable outcome measures, as they are associated with disease status and the presence of co-morbidities, and they are core components of strategic needs assessments for cancer survivors [28]. However, the majority of these interventions lasted for 6 months or more and were resource intensive (for example, personalised materials, highly trained coaches). Although cost-effective analyses are scarce [29], such intensity or duration may render them inappropriate for wide dissemination. Furthermore, these interventions may not be fully applicable to other cancer groups given the differences between long-term treatment effects. Qualitative work with endometrial cancer survivors suggests they highly desire information regarding specific late-treatment effects and advice on healthy eating and physical activity post-treatment and in person [Koutoukidis et al: Attitudes, challenges, and needs about diet and physical activity in endometrial cancer survivors: a qualitative study. Submitted]. Finally, similarities in effectiveness of programs delivered across developed countries are expected. However, both the health systems and the causes of obesity differ between the USA and the United Kingdom [30]. Despite the common focus of such programs on healthy eating and physical activity, these differences might make a program developed in one context less applicable in the other.

A need exists, therefore, to develop effective behaviour change interventions for endometrial cancer survivors that meet an identified need and can be implemented in the cancer care pathway both in terms of length of programme and resource use. Pre-existing programmes for improving diet and physical activity in the general population, which have demonstrated usability within the National Health Service (NHS), may be an untapped resource. These programmes could be adapted to take into account the specific needs and experiences of endometrial cancer survivors while retaining the core components that have made them effective in other populations.

### Aim

The aim of this pilot study is to assess the feasibility of a manualised healthy eating and physical activity programme in endometrial cancer survivors post active treatment.

The main research question is as follows: ‘Is it feasible to design a randomised controlled trial that will assess if the *Shape-up following cancer treatment* programme is more effective than usual care in improving the health-related quality of life of endometrial cancer survivors?’

### Study objectives

#### Primary research objective

The primary research objective is to assess the feasibility of the overall trial procedures.

#### Secondary research objectives

Secondary research objectives are to accomplish the following: (1) to obtain variance estimates for clinical outcome measures to be used in the large-scale RCT that will inform the measurement of the primary outcome for the larger trial and, subsequently, the sample size calculation; (2) to assess willingness of the clinical staff to recruit participants; (3) to assess willingness of eligible participants to be randomised; (4) to examine potential adverse effects of the intervention; (5) to perform a basic economic analysis with the aim to inform the larger trial; (6) to assess reasons for loss to follow-up; and (7) to access the overall acceptability of the intervention.

## Methods/design

The protocol has been prepared according to Standard Protocol Items: Recommendations for Interventional Trials (SPIRIT) [31] and Template for Intervention Description and Replication (TIDieR) guidelines [32]. For completed checklists, see Additional files 1 and 2.

### Preliminary work

The intervention is based on *Shape-up: a lifestyle programme to manage your weight,* an 8-week weight management programme, which aims to help service users learn new behaviours and manage their weight [33, 34]. This manualised healthy lifestyle programme is based on social cognitive theory [35] and control theory [36] and focuses on self-control, self-efficacy, and behavioural relapse prevention. The intervention is under the tier 2 weight management services [37] and in line with NICE guidance on lifestyle weight management services [17] and individual approaches in behaviour change [38]. Currently, a version of original program is successfully being run in two London boroughs as part of the local joint strategic needs assessment [39].

The programme has been renamed (*Shape-up following cancer treatment: a self-help guide to eating well and being active*), and the intervention development process will be separately reported. In brief, the tailored version is focused on healthy eating and physical activity rather than weight loss, a focus based on the lack of strong evidence for the benefits of intentional weight loss in cancer survival outcomes. A stronger focus on resistance, flexibility and balance exercises has been be added, given their benefits in cancer survivors [40, 41]. Furthermore, specific recommendations about radiotherapy and chemotherapy treatment effects have been added to the booklet, such as avoiding high-fat foods or choosing cooked vegetables.

### Study design

The DEUS pilot trial is an 8-week, two-arm, individually randomised, controlled pilot trial comparing the use of the *Shape-up following cancer treatment* programme to usual care. According to MRC guidance for complex interventions [42], this is a Phase 2 feasibility study. Randomisation will be performed with minimisation, using a 1:1 allocation.

### Study setting

Participants will be recruited from two major academic hospitals in London with sufficient caseload of endometrial cancer patients; University College London Hospitals NHS Foundation Trust, and Barts Health NHS Foundation Trust. The intervention program will be delivered at the University College Hospital Macmillan Cancer Support and Information Centre, located in central London.

### Selection of subjects

#### Inclusion criteria

Women aged > 18 years (no upper age limit) who have been diagnosed with endometrial cancer (ICD C54.1) within the previous 36 months will be eligible to take part in the study. They must also be able to understand spoken and written English. The cut-off of 36 months was chosen to account for the duration of treatment and allow for a sufficient pool of survivors for recruitment, so the presence of the teachable moment and the elimination of early major treatment effects can be balanced.

#### Exclusion criteria

Women who meet at least one of the following criteria will be excluded: (1) diagnosed with stage IVB (metastatic) endometrial cancer (any metastasis beyond the pelvis); (2) undergoing active anticancer, and/or palliative treatment; (3) having a second primary cancer; (4) lacking the mental capacity to decide to take part in the study and to participate in it (based on the clinical team’s judgement in accordance with the Mental Capacity Act 2005 Code of Practice 2007); (5) having severe depression (consultant’s judgement based on the DSM-IV criteria); (6) unavailable for longitudinal follow-up assessments; (7) having participated in a professionally delivered weight loss or exercise program during the previous 6 months; (8) having a WHO performance score 3-4 [43]. These criteria comply with all but the disability category in the NICE Equality Impact Assessment for lifestyle weight management services [17].

### Interventions

#### Active intervention

A researcher in nutrition and dietetics (DAK), trained by Weight Concern, who has clinical experience with cancer survivors, will facilitate the *Shape-Up following cancer treatment* sessions following the standardised and scripted manual. An extra trained provider will attend the intervention meetings to aid with facilitation but will not participate in the discussion. S/he will deliver the intervention in case of unpredictable circumstances. In addition to usual care, they will be assigned to groups of eight on a first-come first-served basis to avoid delays in delivering the intervention. These groups will meet for weekly 90-minute sessions for 8 weeks.

The tailored version’s primary focus is on strategies for improving healthy eating and physical activity and brief advice on weight management for those who would like it. Behavioural techniques include self-monitoring of behaviour with the use of food and physical activity diaries, behavioural goal setting, action planning, graded tasks, problem solving, self-reward, and review of behavioural goals. It also provides information about the health consequences and emotional consequences of making dietary and activity changes, pros and cons, behavioural practice, habit formation, reducing exposure to cues for the behaviour, behaviour substitution, distraction, social support (unspecified), demonstration of behaviour (for resistance exercises), instructions on how to perform the behaviour (for resistance exercises), and reframing [44].

The course structure and content are shown in Table 1. The format of the intervention is self-help and peer education. Each week, participants will be asked to read part of the manual in preparation for the following week’s new topic. Participants should set their first SMART goal (Specific, Measureable, Attainable, Relevant, Time-bound) for regular eating after the second session, and the first SMART goal for physical activity after the third session. After each of the subsequent sessions, participants should set at least one eating and one physical activity SMART goal.

**Table 1:** Structure and content of the *Shape-up following cancer treatment* sessions

# Session	Session title	Session content	Approximate time
Session 1	Preparing to Shape-up	Welcome and introduction to the programme	25 min
		Setting ground rules for the group	10 min
		Discussion about previous experience of diet and physical activity changes, how cancer has shaped their eating and activity patterns, and hopes and fears about the programme	15 min
		Motivation for change	10 min
		Break	5 min
		Setting a lifestyle target	10 min
		Information and discussion about the importance of self-monitoring and food diaries	10 min
		Round-up and preparation for next session	5 min
		Take-home message	5 min
Session 2	Keeping to a regular eating pattern	Review: Discussion about self-monitoring and food diaries and goal progress	
		Volunteer-led discussion: Keeping to a regular eating pattern	
		Key learning points: The importance of keeping to a regular eating pattern, the definition of a regular eating pattern, the importance of breakfast, suggestions for goals, and disadvantages of eating regularly.	
		New topic: Goals and rewards	
		Discussion about the principles of goal-setting, group exercise about setting SMART goals, exercise about goal planning, discussion about rewards, and group exercise about non-food rewards	
		Round-up and preparation for next session	
		Take-home message	
Session 3	Physical activity	Review: Discussion about keeping a diary, setting a regular eating goal, goal-setting, rewards, and goal progress	20 min
		Volunteer-led discussion: Physical activity	30 min
		Key learning points: The importance of physical activity for health and wellbeing, the difference between physical activity and exercise, goals to aim for (30 min of moderate physical activity per day and muscle fitness exercises twice a week), and the importance of incremental increase in physical activity levels.	
		Break	10 min
		Setting an activity goal: individual exercise to help them focus on what they need to think about in order to improve their activity levels and group exercise for improving goal-setting skills	15 min
		Round-up, and preparation for next session	10 min
		Take-home message	5 min
Session 4	Eating a balanced diet	Review: Discussion about last week’s topics and creating a physical activity goal and goal progress	20 min
		Volunteer-led discussion: Getting a healthier balance of foods.	30 min
		Key learning points: the five food groups, choosing which foods to make (plenty of whole grains, fruits, and vegetables; moderate amounts from the ‘meat, fish and alternatives’ and ‘milk and dairy’ groups, preferably low-fat; have little amounts of ‘foods high in fat or sugar’ and prefer healthy oils; limit processed meat, and sugary and alcoholic drinks; prefer foods low in salt).	
		Break	5 min
		New topic: Lapses	25 min
		Information, group exercise, and discussion on how to deal with lapses	
		Round-up, and preparation for next session	5 min
		Take-home message	5 min
Session 5	Keeping an eye on food serving sizes	Review: Discussion about last week’s topics, goal progress, and exercise about managing lapses	20 min
		Volunteer-led discussion: Keeping an eye on food serving sizes	50 min
		Key learning points: Each participant brings a weighed portion of some foods, and group members discuss understanding food serving sizes and how many servings they should aim for (the food servings reflect a 2,000 kcal diet for women).	
		Break	10 min
		Round-up and preparation for next session	5 min
		Take-home message	5 min
Session 6	External triggers	Review: Discussion about last week’s topics and goal progress	20 min
		Volunteer-led discussion: External triggers	35 min
		Key learning points: The difference between external and internal triggers, and main strategies for dealing with external triggers	
		Break	5 min
		New topic: Internal triggers	20 min
		Discussion about hunger and cravings, group exercise for the dealing with cravings and the difference between craving and hunger and discussion about fatigue	
		Round-up and preparation for next session	5 min
		Take-home message	5 min
Session 7	Internal triggers	Review: Discussion about last week’s topics, goal progress, and feelings about the group coming to an end	20 min
		Volunteer-led discussion: Internal triggers	40 min
		Key learning points: Definition of internal triggers (hunger, cravings, fatigue, emotions, and unhelpful thoughts) and strategies to deal with those	
		Break	5 min
		New topic: Lapse chains	10 min
		Information about lapse chains and group exercise of putting together a behaviour chain	
		Round-up, and preparation for next session	10 min
		Take-home message	5 min
Session 8	Food labels and the *Shape-up* change plan	Review: Discussion about last week’s topics and goal progress and reviewing earlier areas of the programme	10 min
		Volunteer-led discussion: Food labels	30 min
		Key learning points: The ingredient list and the various names of sugar and saturated fat in food labels, the importance of checking the sugar, saturated fat, and salt content in the labels, and tips for smart shopping	
		Break	5 min
		New topic: The *Shape-up* change plan	20 min
		Information about how to complete a *Shape-up* change plan and individual exercise on filling a mock plan	
		Discussion: Group members discuss their change plan, they re-evaluate their lifestyle target from Session 1, and are given information about the importance of continuing with self-monitoring	20 min
		Round-up, and maintaining changes for the long term	5 min
		Take-home message	5 min

Those who miss sessions will receive standardized e-mails or mail with the content of the session. They will also be asked not to discuss the intervention with fellow patients in an attempt to minimize contamination and avoid leakage of intervention details among patients in the study arms.

### Control group

Participants in the control group will be offered usual care. Quantifying usual care is challenging, but our preliminary qualitative study suggested that lifestyle advice is limited in the current clinical setting [Koutoukidis et al Attitudes, challenges, and needs about diet and physical activity in endometrial cancer survivors: a qualitative study. Submitted]. During the trial, participants will only be contacted for the assessments. After the final follow-up, the researcher will have a 5-minute discussion with them using a standard statement focusing on the link between lifestyle and health consequences and targeting their motivation to improve their health. At that point, they will also receive the ‘Healthy living after cancer’ booklet, a brief self-help manual produced by the World Cancer Research Fund [45].

### Outcome measures

#### Primary outcome measures

The primary outcome measures for the pilot trial are as follows:

1. The recruitment rate.
2. The adherence rate (attendance of the sessions).
3. The retention rate (complete follow-up).

The main criterion to judge the pilot study successful and a large-scale RCT feasible using the recruitment measure is recruiting (consenting) 30 % of the eligible participants (32 participants per 110 estimated to be eligible in each centre during the 6-month recruitment). This target seems reasonable based on our previous experience and similar rates indicated in the literature [46, 47]. The primary measure to be used in the large-scale RCT is projected to be a change in global quality of life as measured by the EORTC Quality of Life Questionnaire (QLQ-C-30). However, the choice of additional primary outcomes will be finalised after taking into account the results of the feasibility study.

#### Secondary outcome measures

1. Clinical outcomes to be used in the large RCT include the following:
  I. Health-related quality of life
  II. Diet quality
  III. Physical activity
  IV. Hand-grip strength
  V. Weight
  VI. Body composition
  VII. Shape-up evaluation questionnaire
  VIII. Healthcare services use
  2. Willingness of clinical staff to recruit participants will be assessed with a short one-to-one interview with the clinicians at the beginning of the third month of recruitment.
  3. The number and type of potential adverse effects of the intervention will be recorded during the intervention and at the follow-up interview (for example, gastro-intestinal complaints from a change in diet).
  4. Costs relevant to recruitment, screening, implementation, and follow-up will be calculated. We will also measure retrospectively the healthcare resource use and cost them at national rates.
  5. Reasons for non-participation and loss to follow-up will be tracked for each participant lost and merged in similar categories.
  6. At 8 and 24 weeks follow-up, a purposive sample (30 %) of participants in each arm will be interviewed to assess their experience of participating in the trial, the acceptability of the intervention and the materials, and their overall experience of the program, including potential facilitators or barriers to adherence. All participants who may dropout will also be approached for an interview. These data will help with the refinement of the intervention.

### Participant timeline

**Fig. 1:**
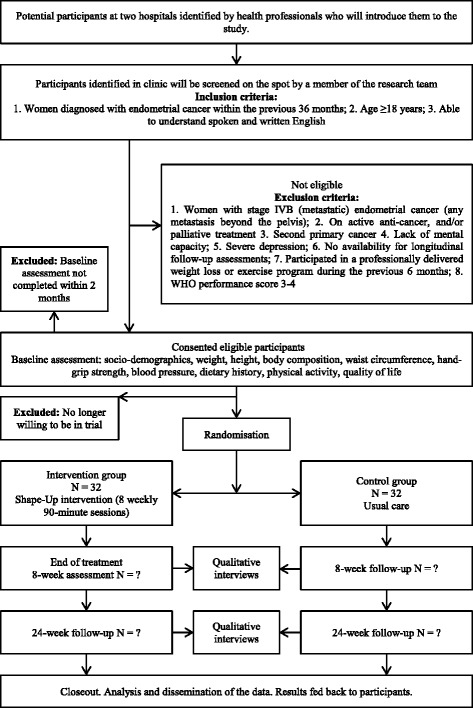
Flow-chart

Figure 1 demonstrates the flow chart of the study. Table 2 shows the assessments at each time point.

**Table 2:** Study assessments at specific time points

			Study Period				
	Staff member	Pre-study screening/consent	Baseline	Allocation	Post-allocation		
Time point			Week -3	Week 0	Week 1	Week 8	Week 24
ENROLMENT							
Eligibility screen	Interviewer	X					
Informed consent	Interviewer	X					
Allocation	Study Coordinator			X			
INTERVENTIONS							
Shape-up	Interviewer						
Usual care	Interviewer						
ASSESSMENTS							
Socio-demographic data	Interviewer		X				
EORTC-QLQ-C30	Interviewer		X			X	X
EORTC-QLQ-EN34	Interviewer		X			X	X
Dietary assessment	Interviewer		X			X	X
Physical activity	Interviewer		X			X	X
Weight	Interviewer		X			X	X
Height	Interviewer		X				
Body composition	Interviewer		X			X	X
Waist circumference	Interviewer		X			X	X
Hand-grip strength	Interviewer		X			X	X
Blood pressure	Interviewer		X			X	X
Shape Up evaluation	Interviewer					*X* ^2^	*X* ^2^
Control group input	Interviewer						X^3^
Qualitative interviews	Interviewer					X	X
Health care resource use	Interviewer						X
EQ5D-3 L	Interviewer		X			X	X
Serious adverse report form	Study Coordinator	As needed throughout the protocol					

^1^Closeout for the control group will be at week 40 after receiving the intervention

^2^Only the intervention group

^3^Only in the control group

### Sample size

Although this is a feasibility study, a sample size has been specified for examining the recruitment rate, using A’Hern’s approach for one-stage phase II trials [48]. Regarding recruitment, a success rate of approximately 30 % or more would be desirable for the trial to be considered feasible. A success rate of 15 % or less would be unacceptable. The trial will test the null hypothesis H_0_ that recruitment is ≤ 15 % against the alternative hypothesis H_1_ that recruitment is ≥ 30 %. With a 5 % level of significance and 90 % power, 64 participants are needed so that we can estimate whether the percentage of participants with successful recruitment is ≤ 15 % or ≥ 30 %. If we can recruit 15 or more participants, we can reject the null hypothesis.

A trial of 64 (32 per arm) will be sufficient to test the above hypothesis and allow decisions to proceed to a Phase III trial. It will also allow for rich feedback from the participants to be used for the optimisation of the procedures and the materials. Lastly, it will allow a certain degree of precision in calculating standard deviations for the secondary outcomes that will be the key design parameters for the main study [49].

### Recruitment

Potential participants will be recruited from outpatient clinics at the two hospitals. They will be identified and approached by a member of the clinical team. Bright colour reminders will be attached at the cover of the patient notes before their appointment to enhance consultants’ engagement with recruitment. After initial screening from the clinician, and if the patient is willing to hear about the study, following verbal consent, the patient will be introduced to the researcher attending the clinic for final screening and a detailed discussion of the study. All participants will need to consent for themselves following standard procedures (Additional file 3). We expect to recruit on average 1.07 participants per week per hospital during the 30-week recruitment period to reach our target.

The clinical teams in the two hospitals will also identify potential participants who have been treated in the two recruitment sites but have been followed up at local sites. Following the General Practitioner’s verification that the participants are alive, invitation letters signed by the consultant will be sent to these women, along with the participant information sheet, an opt-in form, the barriers to participation survey [50], and a business reply envelope.

### Assignment of interventions

#### Sequence generation

Consented participants will be individually randomised with a 1:1 allocation to receive either the intervention or usual care through minimisation [51]. The two stratified variables are age (cut-off: 61 years) and obesity (BMI cut-off: 30 kg/m^2^), as these are strong prognostic factors of all clinical outcome measures. The age cut-off was chosen as this is the median age of diagnosis for endometrial cancer [52] and the BMI cut-off is the WHO cut-off to classify obesity.

The allocated treatment will be determined using MinimPY software run by RJB. Initially, the first participant will be randomly allocated. For each of the following participants, allocation will be based on the imbalance scores, calculated as a function of current allocations after a hypothetical allocation of the new participant in each study arm. The new participant will be allocated to the arm with the least imbalance score [53]. A 20 % random element will be included in the algorithm [51].

### Allocation concealment mechanism

The researcher (RJB) who generates the allocation sequence will keep the sequentially numbered opaque sealed envelopes. The rest of the team will have no physical access to them. The researcher will maintain no contact with the rest of the group about the allocation concealment until enough participants are allocated for a *Shape-up following cancer treatment* group to run.

### Implementation

Following the baseline assessment, DAK will feed back to RJB the BMI and the age of the recruited participant in a randomisation form in a sealed opaque envelope. The latter will run the algorithm and allocate the participant. This process will continue until enough participants are allocated in both groups to run a *Shape-up following cancer treatment* group. Apart from RJB, all research team members will be blinded to group allocation until a group can be run (for example, the first 16 participants have been randomised). At that point, participants will also be notified in which group they have been allocated.

### Blinding

Due to the nature of the intervention, neither participants nor the researchers delivering the intervention can be blinded. The independent trained assessor for the 8-week follow-up will be blinded to treatment allocation, and requests have been made of the participants not to disclose their allocation treatment. The assessor of the 24-week follow-up (DAK) will not be blinded given resource constraints.

### Data collection, management and analysis

#### Data collection methods

Participants will visit the laboratory for three 90-min one-to-one assessments. They will complete the widely used, reliable and validated European Organization for Research and Treatment of Cancer Quality of Life Questionnaire (QLQ-C30) [54] and Endometrial Cancer Module (QLQ-EN24) [55]. We will use the Stanford 7-Day Physical Activity Recall [56], a 15-min interview-based tool, to assess physical activity, which has shown acceptable reliability and validity [57] and is responsive to change.

Dietary intake will be assessed with one weekday 24-hour dietary recall using the ASA24 web-based tool [58]. Nutrition and statistical software will be used to calculate how well the participant’s diet fits to recommended healthy eating patterns. Our previous piloting of the assessment indicated acceptability and feasibility. Diet quality will be calculated by the Alternative Healthy Eating Index (AHEI) score, assessing how well a diet fits to recommended healthy eating patterns and being a strong predictor of survival [59]. An updated version of the DINE questionnaire [60], together with questions about fruits and vegetables, will supplement the 24-hour dietary recall data.

Weight to the nearest 0.1 kg and body composition will be assessed using MC980 multi-frequency segmental body composition analyser. Using standardised protocols, standing height will be assessed to the nearest 0.1 cm, and handgrip strength, using a handgrip dynamometer. Waist circumference will be measured to the nearest 0.1 cm [61]. Blood pressure will be measured using an automated sphygmomanometer with the participant seated comfortably for 5 minutes before measurement and the arm supported at the level of the heart. All measurements will be taken twice and averaged for analysis. Participants will also complete six socio-demographic questions. Researchers will be receiving standardised training with all measurements.

Participants will also complete the *Shape-up* questionnaire at all time points; ten items in a five-point Likert scale (from ‘Strongly disagree’ to ‘Strongly agree’) that reflect the overall goals of the programme (for example, I am in control of my food portion sizes, I can set effective eating and activity goals and work towards them). At the end of the program, the intervention-arm participants will be given an 18-item evaluation form [62] to complete at home and return by post. At the 24-week follow-up, the control arm will complete two more questions assessing the input about diet and physical activity they received from external sources, to allow an assessment of contamination. At the last follow-up, all participants will also complete a healthcare resource using a six-item questionnaire [63]. Quality-of-life adjusted years (QALYs) will be assessed with the validated six-item EQ5D-3 L [64].

After they have taken enough time to decide about their participation, individuals will be asked their reason(s) for non-participation [50]. The same questions and prompts will be asked of participants who decide to withdraw from the study. Participants may withdraw from the study for any reason at any time. However, we will make every reasonable effort to follow the participants for the entire study period. If a follow-up appointment in the laboratory is not possible after three consecutive contact attempts, a researcher will request to visit the participants at home for the interview or undertake this by telephone. Participants will not be aware of this option beforehand to maximise the chances for having the physical measurements.

### Data management

This study has been registered for Data Protection at UCL Records Office (Reference: Z6364106/2014/12/14). Standard procedures following Data Protection Act 1998, the NHS Code of Confidentiality, and Good Clinical Practice will be implemented throughout the study.

### Statistical methods

In addition to recruitment rate, the study is also examining adherence rate and retention rate (complete follow-up). Adherence is defined as the proportion of engaged participants attending at least one of the last three sessions of the intervention. Engaged participants are those who have attended at least two sessions of the intervention. Best practice guidance suggests that programmes should be commissioned if at least 60 % of participants are likely to adhere [37]. A success rate approximately of 85 % or more would be desirable. That means that 85 % or more of the engaged participants in the intervention group will attend at least one of the last three sessions of the intervention. A success rate of 60 % or less would be unacceptable. The trial will test the null hypothesis H_0_ that adherence is ≤ 60 % against the alternative hypothesis H_1_ that adherence is ≥ 85 %. With a 5 % level of significance and 90 % power, 27 participants are needed so that we can estimate whether the percentage of participants with successful adherence is ≤ 60 % or ≥ 85 %. If 21 or more participants have a successful adherence, we can reject the null hypothesis.

Regarding retention (attendance of both follow-up sessions) rate, a success rate of approximately 75 % or more would be desirable. A success rate of 60 % or less would be unacceptable. The trial will test the null hypothesis H_0_ that complete follow-up is ≤ 60 % against the alternative hypothesis H_1_ that complete follow-up is ≥ 75 %. With a 5 % level of significance and 80 % power, 62 participants are needed so that we can estimate whether the percentage of participants with complete follow-up is ≤ 60 % or ≥ 75 %. If 44 or more participants have a complete follow-up, we can reject the null hypothesis.

Recruitment, adherence and retention rates will be reported as proportions with 95 % CIs. The target lower 95 % confidence limit for the following outcomes are 15 % or more, 60 % or more, and 60 % or more for recruitment, adherence, and retention, respectively.

Continuous variables will be reported by descriptive statistics (non-missing sample size, mean, standard deviation, median, maximum and minimum). Categorical variables will be summarised using frequencies and percentages. Analysis of covariance (ANCOVA) will be used to compare the intervention arm against the control arm in an exploratory way, as the study is not powered to detect differences. All participant data will be analysed using the intention-to-treat strategy [65]. Adjustment for BMI and age will be performed with linear regression in continuous outcomes and logistic regression in binary outcomes. Missing outcome data will be imputed using multiple imputations. Reasons for missing data will be documented, and missing data will be quantified. The Statistical Package for Social Sciences (SPSS, Chicago, IL) version 21 will be used for the whole data analysis. Adverse events will be reported descriptively. The level of statistical significance will be set at 5 % for the primary outcome measures. We will also calculate intra-class correlation coefficients (ICCs) to measure clustering within groups and k coefficient of variation between groups.

Qualitative data will be analysed using thematic analysis. Two interview transcripts will be independently coded by two researchers. These lists will be discussed and amended between researchers upon agreement until relevant themes are identified. DAK will insert the code lists into NVivo software version 10 (QSR International Pty Ltd, 2014). NVivo version 10 (QSR International Pty Ltd, 2014). Two random transcripts will be recoded by an independent researcher to ensure consistency.

### Monitoring

#### Data monitoring

Given the short length of the intervention, the low risk of harm (see below) and the short follow-up of the intervention, an external Data Monitoring Committee will not be needed and an interim analysis will not be performed. Nonetheless, the researchers recruiting, implementing, and assessing the intervention will update the research team monthly about the study progress.

### Harms

*Shape-up following cancer treatment* is a very low intensity intervention that should be suitable for most people with health conditions, such as diabetes, heart failure, and high blood pressure. Observed changes are unlikely to be associated with unintended or adverse effects [17]. The proposed modifications to lifestyle, that is, dietary and physical activity changes, are in accordance with published guidelines for cancer survivors [9]. All potential adverse effects and unintended effects of the intervention will be reported.

### Auditing

The sessions will be audiotaped and the recording will be coded against the *Shape-up following cancer treatment* Facilitators manual for assessing intervention delivery and treatment receipt by a researcher experienced in health psychology and behaviour change [66]. The group facilitator (DAK) will also audio record a short debriefing after each session. RJB will randomly perform undisclosed site visits in two assessments and one intervention session to assess protocol fidelity. The *Shape-up* evaluation form, which includes a self-assessment of the gained skills and an evaluation of the facilitator, will supplement fidelity assessment. Results will inform improvements in protocol fidelity.

### Ethics and dissemination

#### Research ethics approval

The study protocol and documents have been reviewed and approved by the relevant sponsor and National Research Ethics Service Committee London - City Road and Hampstead (Reference: 15/LO/0154).

### Protocol amendments

Potential protocol modifications will be formally approved by the REC before being implemented. The amendments will be communicated to the trial registries and outlined at the study dissemination.

### Post-trial care

#### Archiving

Study-related documents will be archived at UCL and each participating site at the end of the study for 20 years and in line with all relevant legal and statutory requirements.

### Dissemination policy

The period for study dissemination will be kept to the minimum possible. The primary papers will report the primary outcome measures. The results will be disseminated regardless of the magnitude or direction of effect. All investigators will be authors of future publications with authorship eligibility to follow international guidelines [67]. The study results will also be disseminated to the clinical teams in the participating centres and to the participants. A completely de-identified dataset will be disseminated to a relevant data archive for sharing purposes no later than 3 years after the study closeout.

### Roles and responsibilities

#### Trial Management Group

DAK will be responsible for the daily monitoring and management, reporting directly to AL. AL has overall responsibility for the project. The two study site CIs (AL, RM) will oversee the identification of potential participants. RJB and TMK will provide expert advice during the study and the analysis and interpretation of the results. Professor Steve Morris will provide advice regarding the health economic aspects. RJB will be responsible for the intervention assignment and auditing.

#### Trial Steering Committee

An external Trial Steering Committee that will meet at regular intervals during the study will oversee the trial. The committee, which is chaired by Professor Allan Hackshaw, will include two other independent members, the two site PIs (AL and RM), the trial co-investigators and a lay representative.

## Discussion

In the UK, only a few studies have demonstrated the feasibility of interventions for lifestyle behaviour change in cancer survivors [68, 69]. None of them, however, has involved the growing population of endometrial cancer survivors. This feasibility trial will inform a larger lifestyle trial in cancer survivors to test if the program can help survivors to improve their quality of life. The outcome of this pilot study will be translated as (a) a feasible study that should be continued without modifications, (b) a feasible study with close monitoring that should be continued without modifications, (c) a feasible study with modifications in the protocol or (d) a non-feasible study. The study has the potential not only to help cancer survivors improve their well-being but also to help NHS reduce its cost by potential reduction of the use of its services as survivors will lead a healthier lifestyle.

The current intervention is in accordance with the National Cancer Survivorship Initiative, which envisages a sustainable personalised lifestyle support for cancer survivors with them playing an active part in the decision-making in addition to research on patient-reported outcomes [70]. If proven effective, we hope that *Shape-up following cancer treatment* will be disseminated nationally as a low-cost, self-help, group program. The manualised format and the facilitator’s guide allow for standardised training for facilitators that could be non-healthcare professionals, accurate replication and evaluation across settings.

### Trial status

The trial is currently recruiting participants.

## Additional file

Additional file 1: SPIRIT statement. (DOC 121 kb) Additional file 2: TIDieR statement. (DOC 60 kb) Additional file 3: Consent form. (DOCX 31 kb)

## Abbreviations

BMI: body mass index
EORTC: European Organisation for Research and Treatment of Cancer
ICD: International Classification of Diseases
NHS: National Health Service
NICE: National Institute for Health and Care Excellence
PI: Principal Investigator
RCT: Randomised Clinical Trial
SMART: Specific, Measureable, Attainable, Relevant, Time-bound
UK: United Kingdom
USA: United States of America
