# Erratum to: Diet and exercise in uterine cancer survivors (DEUS pilot) - piloting a healthy eating and physical activity program: study protocol for a randomized controlled trial

**DOI:** 10.1186/s13063-017-1776-z

**Published:** 2017-01-18

**Authors:** Dimitrios A. Koutoukidis, Rebecca J. Beeken, Ranjit Manchanda, Matthew Burnell, M. Tish Knobf, Anne Lanceley

**Affiliations:** 1 Department of Women’s Cancer, EGA Institute for Women’s Health, University College London, London, UK; 2 Health Behaviour Research Centre, Department of Epidemiology & Public Health, University College London, London, UK; 3 Department of Gynaecological Oncology, Barts Health NHS Trust, Royal London Hospital, London, UK; 4 Barts Cancer Institute, Queen Mary University of London, London, UK; 5 Acute Care/Health Systems Division, Yale University School of Nursing, New Haven, CT USA

## Erratum

Upon publication of the article [1], it was noticed that Table 1 was missing some times in the 4th column of ‘Session 2’. This information has now been included in this erratum.

**Table 1:** Structure and content of the Shape-up following cancer treatment sessions

Session 2	Keeping to a regular eating pattern	Review: Discussion about self-monitoring and food diaries, and goal progress	20 min
		Volunteer-led discussion: Keeping to a regular eating pattern Key learning points: The importance of keeping to a regular eating pattern, the definition of a regular eating pattern, the importance of breakfast, suggestions for goals, disadvantages of eating regularly.	40 min
		Break	5 min
		New topic: Goals and rewards Discussion about the principles of goal-setting, group exercise about setting SMART goals, exercise about goal planning, discussion about rewards, and group exercise about non-food rewards	15 min
		Round-up, and preparation for next session	5 min
		Take home message	5 min
