# Supplementary material for: Diet and exercise in uterine cancer survivors (DEUS pilot) - piloting a healthy eating and physical activity program: study protocol for a randomized controlled trial
Source: Trials. 2016 Mar 10;17:130. doi: 10.1186/s13063-016-1260-1 (PMC4785620; doi:10.1186/s13063-016-1260-1)
Supplement: Additional file 3: — Consent form. (DOCX 31 kb) [file 13063_2016_1260_MOESM3_ESM.docx]

**Appendix 3: Consent form**

[Hospital & Universtity logo]

Study Number: 170437

Patient Identification Number for this study: D E U S p _

**CONSENT FORM**

Title of Project: DEUS pilot study: Diet and Exercise in Uterine Cancer Survivors (Student Study)

Name of Researcher: **Dimitrios Koutoukidis**

Please initial all boxes

1. I confirm that I have read and understand the information sheet dated **17/03/15 (version 3.0)** for the above study. I have had the opportunity to consider the information, ask questions and have had these answered satisfactorily.
2. I understand that my participation is voluntary and that I am free to withdraw at any time without giving any reason, without my medical care or legal rights being affected.
3. I understand my medical records will be reviewed by relevant researchers in this study.
4. I understand that relevant sections of my medical notes and data collected during the study may be looked at by individuals from University College London, from regulatory authorities or from the NHS Trust, where it is relevant to my taking part in this research. I give permission for these individuals to have access to my records.
5. I agree to have the sessions and telephone interviews audio-recorded.
6. I understand that I will not benefit financially if this research leads to the development of a new manual about cancer.
7. I agree that my GP will be informed about my participation in the study.
8. I agree to take part in the above study.

Name of Participant Date Signature

Name of Person Date Signature

taking consent.
